# *Ceratobasidium* sp. is associated with cassava witches’ broom disease, a re-emerging threat to cassava cultivation in Southeast Asia

**DOI:** 10.1038/s41598-023-49735-5

**Published:** 2023-12-15

**Authors:** Ana M. Leiva, Juan M. Pardo, Warren Arinaitwe, Jonathan Newby, Pinkham Vongphachanh, Khonesavanh Chittarath, Samoul Oeurn, Le Thi Hang, Alejandra Gil-Ordóñez, Rafael Rodriguez, Wilmer J. Cuellar

**Affiliations:** 1 Virology and Crop Protection Laboratory, Cassava Program, International Center for Tropical Agriculture (CIAT), Crops for Nutrition and Health Research Area, The Americas Hub, Km 17 Recta Cali, 763537 Palmira, Colombia; 2 Crops for Nutrition and Health, International Center for Tropical Agriculture (CIAT), Cassava Program Asia Office, P.O. Box 783, Vientiane, Lao PDR; 3 Plant Protection Center (PPC), Department of Agriculture, Ministry of Agriculture and Forestry, P.O. Box 811, Vientiane, Lao PDR; 4 Plant Protection Sanitary and Phytosanitary Department, General Directorate of Agriculture (GDA), Phnom Penh, 120406 Cambodia; 5 Plant Protection Research Institute (PPRI), Duc Thang Bac Tu Liem, Hanoi, 100000 Vietnam

**Keywords:** Fungi, Pathogens, Microbiology, Molecular biology, Metagenomics

## Abstract

Cassava witches' broom disease (CWBD) is a devastating disease of cassava in Southeast Asia (SEA), of unknown etiology. Affected plants show reduced internodal length, proliferation of leaves and weakening of stems. This results in poor germination of infected stem cuttings (i.e., planting material) and significant reductions in fresh root yields and starch content, causing economic losses for farmers and processors. Using a metagenomic approach, we identified a fungus belonging to the *Ceratobasidium* genus, sharing more than 98.3–99.7% nucleotide identity at the Internal Transcribed Spacer (ITS), with *Ceratobasidium theobromae* a pathogen causing similar symptoms in cacao. Microscopy analysis confirmed the identity of the fungus and specific designed PCR tests readily showed (1) *Ceratobasidium* sp. of cassava is strongly associated with CWBD symptoms, (2) the fungus is present in diseased samples collected since the first recorded CWBD outbreaks in SEA and (3) the fungus is transmissible by grafting. No phytoplasma sequences were detected in diseased plants. Current disease management efforts include adjustment of quarantine protocols and guarantee the production and distribution of *Ceratobasidium*-free planting material. Implications of related *Ceratobasidium* fungi, infecting cassava, and cacao in SEA and in other potential risk areas are discussed.

## Introduction

Throughout Southeast Asia (SEA), cassava (*Manihot esculenta* Crantz) has traditionally been recognized as a food security crop for poor and vulnerable communities, being the second most important crop after rice in this region^1^. The crop currently provides a source of livelihood for millions of smallholder upland farmers in Mainland Southeast Asia linked to the multi-billion-dollar cassava starch industry, livestock feed sector, and ethanol (industrial and biofuel) industry. The interlinked regional cassava economy has expanded rapidly in recent years because of strong global derived demand and improved infrastructure, leading to high farm-gate prices and attractive economic returns for rural households^2^.

Cassava witches' broom disease (CWBD) was first reported in the Pacific Islands of Wallis and Futuna^3^, and remained largely unnoticed in SEA until 2010, when high incidences and significant fresh root yield reductions were reported in cassava fields of Vietnam, Cambodia, and Thailand^4–6^. Latest reports show that the impact of the disease is extended also in Lao PDR, Myanmar^7^ and the Philippines^8^. In these reports, *Candidatus* phytoplasma asteris and luffae were identified in some of the affected plants. However, the percentage of diseased plants that are positive to phytoplasma (as detected by nested PCR and confirmed by sequencing) is generally not included^4–8^ and on the contrary high percentages of false positives have been reported^9^. So far, there is no reliable molecular test for CWBD diagnostics.

The characteristic symptoms of CWBD include dwarfism and proliferation of weak, spindly sprouts on the cassava stems, resulting in the formation of brooms, hence the name. Cassava stems then develop short internodes, and vascular necrosis along the affected parts^3,9^. Although such symptoms are identified with phytoplasma infections^10,11^, other pathogens can also induce witches’ broom symptoms. For example, cacao witches’ broom disease occurring in the Americas, is caused by the basidiomycetous fungus *Moniliophthora perniciosa*; it induces a disorganized proliferation of the infected vegetative meristems of axillary shoots, which in time become necrotic, forming a structure named ‘dry broom’^12^. In fact, an accumulation of dry leaves is commonly observed at the bottom part of the broom in cassava plants with severe symptoms of CWBD^9^. Due to the significant effect that CWBD has on stem development, production of planting material (stem-cuttings) is significantly limited, forcing farmers to acquire stakes from other sources and therefore increasing the risk of introducing and moving around additional pathogens through informal seed exchange networks in the region^13^.

In addition to a lack of knowledge on the causal agent, management of CWBD in SEA has been hindered by a late development of CWBD symptoms in the field which complicate positive selection^9^, and the presence of co-infecting transboundary diseases such as cassava mosaic disease (CMD)^14^. As with CMD, at low incidence, extension advice has focused on farmers conducting positive and negative selection of harvested stems based on visual symptom, for subsequent replanting within their own fields^15^. This is a challenging situation considering that popular cassava genotypes such as KU50 and Rayong5, show tolerance to CMD but are susceptible to CWBD^9^. Furthermore, the recent historically high cassava root prices and the expansion of CMD has seen considerable movement in cassava stems throughout the region (including across national borders) as farmers expand the production area and seek to access pathogen-free stem cuttings^13^. In the absence of a certified pathogen-free seed system, the region continues under constant threat from CWBD emergence^8,9^, reaching incidence levels where positive–negative selection strategies are becoming less viable, and the disease anticipated to lead to significant productivity declines and loss of economic value in forthcoming seasons.

Here, starting with a metagenomics approach, we report the discovery, occurrence, graft transmission and association between another basidiomycetous fungus, *Ceratobasidium* sp., and CWBD. Interestingly, phylogenetic analysis of the internal transcribed spacer (ITS) identifies *Ceratobasidium theobromae*^16^, the causal agent of vascular-streak dieback (VSD) of cacao in SEA^17^, as one of the closest related fungi. Common symptoms of VSD and CWBD include vascular necrosis and successive leaf drop and axillary bud development, which in the case of cacao gives branches a “broomstick” appearance^18^. Implications of related *Ceratobasidium* fungi, infecting cassava and cacao in SEA and in other potential risk areas are discussed. A robust molecular diagnostic test for CWBD is also described.

## Results

### Lower diversity indexes and a dominance of *Ceratobasidium* sp. characterize CWBD-affected plants

Three diseased plants and three healthy plants from the same field and of the same variety were sequenced. Ninety percent of the total reads obtained complied with the quality parameters needed to continue the analyses. The median number of reads obtained per sample was 216.232 millions (minimum: 196.515 million; maximum: 250.534 million), and 97.39% of them were mapped to the cassava reference genome. The remaining reads (2.61%) produced from 1401 to 35,765 assigned contigs post assembly process, with a median N50 of 707.5. These results are summarized in Table 1.

**Table 1:** Summary of results from Illumina sequencing after filtering the quality of healthy samples (S1-S3) and samples showing clear symptoms of CWBD (S4-S6).

Sample code*	Total reads	% Reads mapped to cassava	# Reads unmapped	No. of assigned contigs	Avg size (bp)	N50 (bp)	*Ceratobasidium* contigs
S1	196,515,051	97.95	4,514,055	1401	679	726	0
S2	250,533,683	98.48	4,184,629	1839	650	689	0
S3	243,291,842	97.93	5,506,137	3596	613	635	0
S4	214,214,660	98.20	4,232,419	26,668	674	761	16,712
S5	218,249,943	98.51	3,581,978	9427	567	587	5528
S6	211,118,163	93.25	15,730,655	35,765	992	3754	19,734

Only samples S4-S6 produced contigs matching *Ceratobasidium* sequences reported in GenBank. None of the samples produced contigs matching phytoplasma sequences.

**Figure 1:**
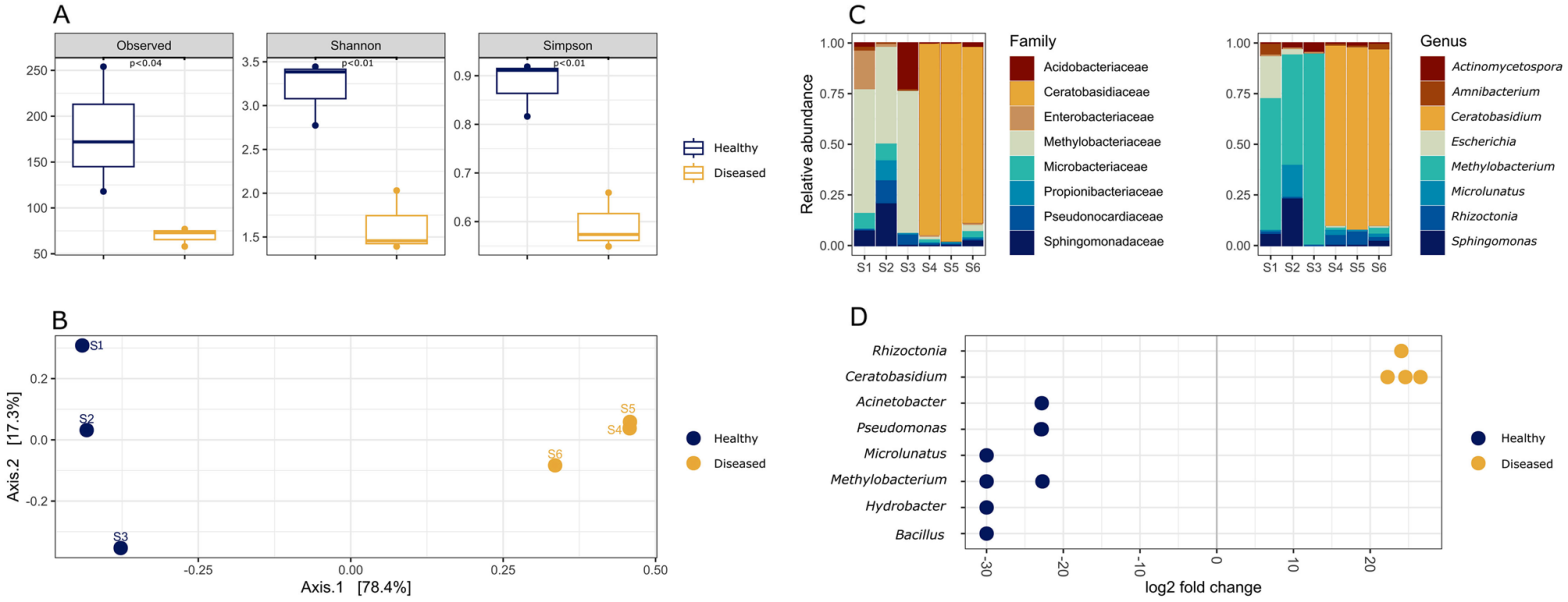
Metagenomic analysis of cassava plants with and without CWBD. (**A**) Alpha diversity indexes of healthy (blue) and diseased plants (yellow), including observed richness, Shannon and Simpson indexes. Box plots show significant differences (p < 0.05) according to the Wilcoxon non-parametric test. (**B**) Principal coordinate analysis (PCoA) with Bray–Curtis distance using OTUs abundance. (**C**) Relative abundance of different taxa identified in healthy (S1-S3) and diseased (S4-S6) cassava plants. Bar plots show the top 8 abundant classifieds within family and genus taxa. normalized by sequencing depth of microbial communities detected in diseased (yellow) and healthy (blue) plants. (**D**) Log-transformed ratio of the relative abundance of 8 genera of OTUs found in diseased (yellow) and healthy (blue) plants estimated by DESeq2.

The three alpha diversity measures indicate that the microbial communities in healthy plants are significantly richer than those in diseased plants. According to the results, healthy plants contained a mean number of species-level Operational Taxonomic Units (OTUs) of 181.3 in comparison with a mean number of 69.3 in diseased plants. The Shannon index in healthy plants had a mean value of 3.19, while the mean value in plants with CWBD was 1.62. The Simpson index also showed a mean value of 0.876 for healthy plants, versus the mean value of 0.586 for diseased plants. The p-values show that these differences are statistically significant, with p-values lower than 0.05 (p < 0.04 for the number of observed species-level OTUs and p < 0.006 for the Shannon index). Additionally, the Simpson index also showed a significant difference between the two groups (p < 0.0034) (Fig. 1A). The difference in composition of microbial communities between healthy and diseased plants is also appreciable in the PCoA, where the normalized abundance of OTUs accounts for 78.4% of the variation between the two groups according to Bray–Curtis distance (Fig. 1B). Likewise, the taxonomic assignations showed clear differences between samples from healthy and diseased plants at both family and genus levels. The most abundant family in diseased plants was *Ceratobasidiaceae*, accounting for 77.5% of assigned contigs. In contrast, the family with the highest number of assigned contigs in healthy plants, *Methylobacteriaceae*, accounted for only 8.7% of assigned contigs (Fig. 1C). The dominance of genus *Ceratobasidium* coincides with the significantly more abundant OTUs detected with the Wald test (Fig. 1D). It is noteworthy, there was not a single contig assigned to *Ceratobasidium* genus in any of the healthy samples (Table 1).

### *Ceratobasidium* sp. associated with CWBD shares characteristic features similar to *C. theobromae*, the causal agent of vascular-streak dieback of cacao

**Figure 2:**
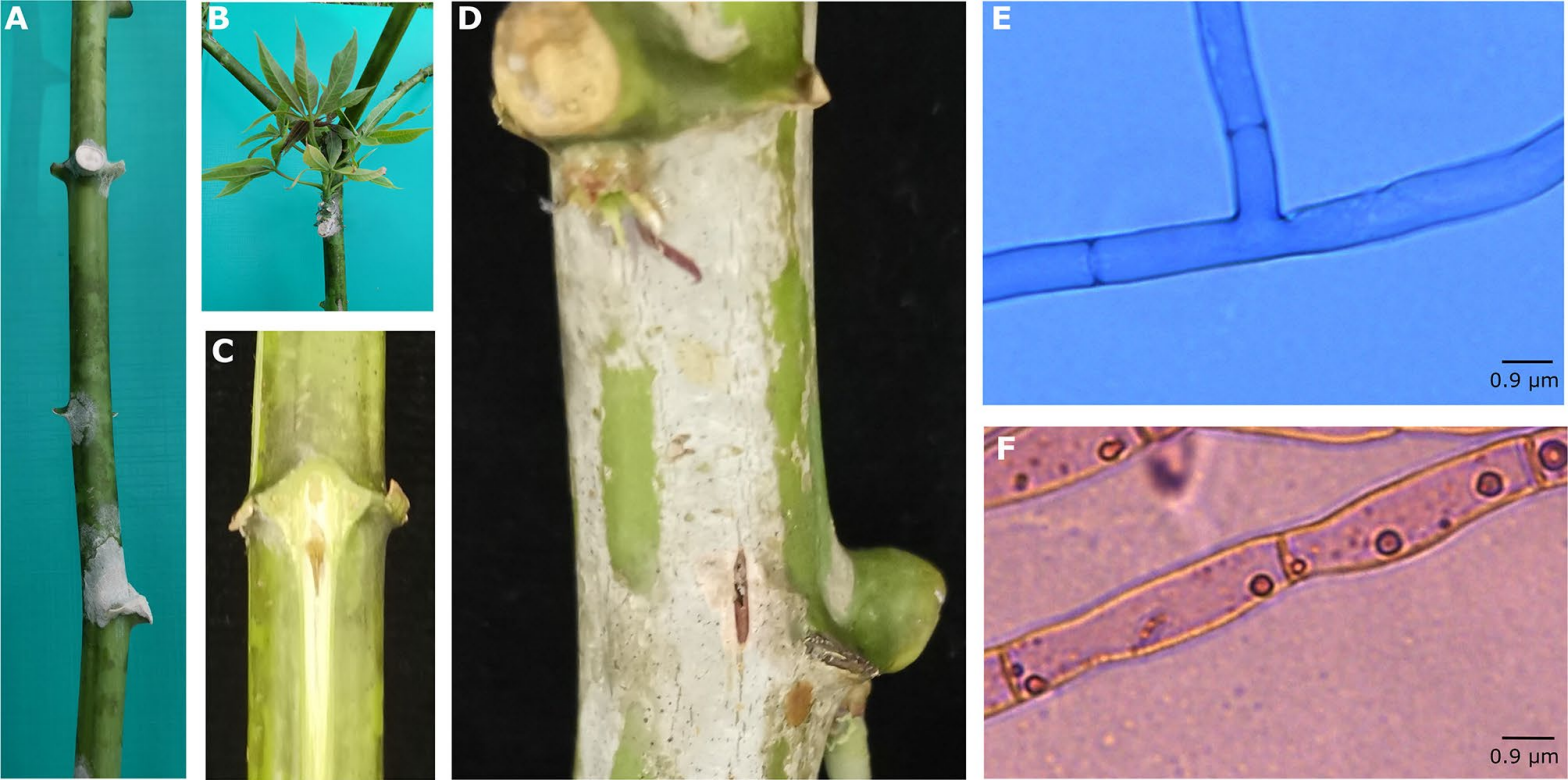
Symptoms associated with *Ceratobasidium* sp. and microscopy observations of the fungus isolated from cassava. Symptoms observed in cassava KU50 with CWBD, both in the field on 12-month-old plants and after graft transmission. Similar symptoms have been reported for VSD caused by *C. theobromae* in cacao^18^. (**A**) Cotton like mycelium growing around the base of petioles. (**B**) Detail of the CWBD symptoms coming out from buds showing fungal-like growth. (**C**) When slicing the petiole base, discoloration (dark central part) is observed exclusively in plants infected with CWBD. (**D**) An enlarged view of the basal bud in (**A**), showing stem splitting symptoms, which are also associated with *C. theobromae*. (**E**) At a magnification of 1000x, the presence of a septa in every branching structure is visible, emerging at a near right angle. (**F**) The bi-nucleate cells hallmark, of members of the genus *Ceratobasidium*.

Cultivating *Ceratobasidium* sp. has proven to be a challenge due to its classification as a fastidious microorganism^16^. The current protocol enabled the isolation of *Ceratobasidium* sp. from symptomatic tissues observed in field-cultivated cassava affected by CWBD. The cotton-like mycelium and the symptoms observed in affected cassava stems and axillary buds looked like those reported for VSD caused by *C. theobromae* in cacao^18^ including the discoloration of vascular tissue and stem splitting (Fig. 2A–D). Isolation included frequent subcultures to delay the growth of faster-growing contaminants, which limited the maintenance period of *Ceratobasidium* sp. pure cultures. Observations under the microscope of isolated hyphae revealed distinct characteristics consistent with members of genus *Ceratobasidium* sp. such as mycelium branching structures emerging at a near right angle, the presence of septa in every branching structure and the bi-nucleate cells hallmark of members of the genus *Ceratobasidium,* which single them out from those in the *Rhizoctonia* group (Fig. 2E and F).

**Figure 3:**
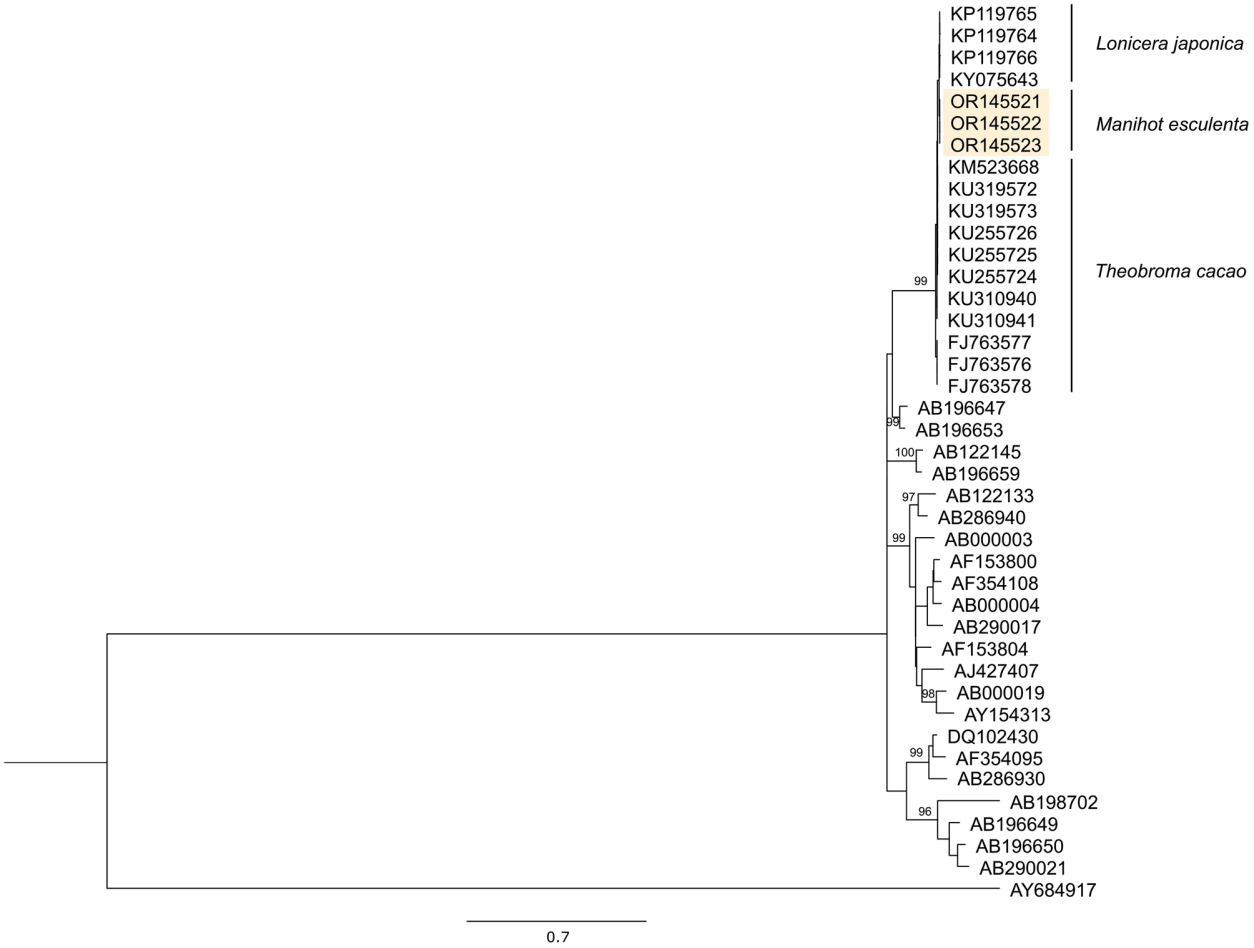
Relationship of *Ceratobasidium* from cassava with isolates causing vascular necrosis in cacao and honeysuckle. *Ceratobasidium* sp. ITS sequences isolated from *M. esculenta* of Cambodia (OR145521), Vietnam (OR145522) and Lao PDR (OR145523) (yellow shading) grouped between ITS sequences isolates from *T. cacao* and *L. japonica*. The tree was constructed using maximum-likelihood method base on GTR + G model (1000 replicates) with related sequences available in GenBank. The bootstrap value for the branch is displayed only when it exceeds 90%. The average nucleotide identity in the compared regions (ITS) of *Ceratobasidium* sequences isolated from cassava and cacao is above 98.3–99.7%. The evolutionary distances were computed using the Maximum Likelihood method. All positions containing gaps and missing data were eliminated (there were 556 positions in the final dataset).

Using the same isolation protocol, we successfully amplified the Internal Transcribed Spacer (ITS) region from samples collected in Cambodia, Vietnam and Lao PDR by PCR. We obtained bands of the expected size (580 bp) sharing more than 98.3–99.7% nucleotide identity with isolates of *C. theobromae*. Phylogenetic analysis readily clustered these sequences in a clade (bootstrap value 99) together with isolates from *Lonicera japonica* and *Theobroma cacao* (Fig. 3) (Supplementary Table 1).

### *Ceratobasidium* sp. from cassava is associated with CWBD in Southeast Asia

**Figure 4:**
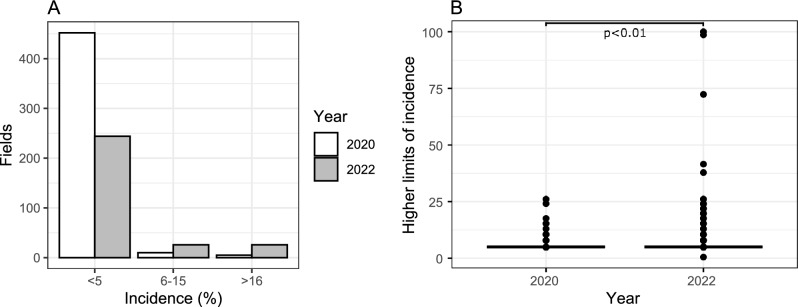
Changes in disease incidence in Southeast Asia. (**A**) CWBD field-incidence intervals observed in SEA during surveys performed in 2020 (white bars) and in 2022 (gray bars). The surveys were organized following a finite population sampling approach with 60 observations per hectare (see materials and methods) on the same locations in both years. (**B**) Box plots showing significant differences (p < 0.05), according to the Wilcoxon non-parametric test. We used upper limits incidence values for 2020 and 2022 as calculated per field (hectare) surveyed. An interactive map of CWBD reports is available at https://pestdisplace.org/embed/news/map/disease/3^15^.

Our latest survey carried out in 2022 showed an increase in the incidence of the disease in Lao PDR, Vietnam and Cambodia in comparison with the results from the 2020 survey. This difference is significant and suggests a re-emergence of the disease. The number of fields with an incidence rate between 5 to 15% has surged by 2.6 times, while the number of fields with incidences exceeding 16%, has skyrocketed by 5.2 times in 2022; in some fields, we have already detected incidences exceeding 50% (Fig. 4, Supplementary Table 2), a situation not observed since 2014^7^. The symptoms recorded were similar across all countries, with stem vascular necrosis being observed in all locations.

To assess whether *Ceratobasidium* sp. isolated from cassava is associated with CWBD in the field, we designed and evaluated three primer sets. First, we assessed the total DNA extracted from a set of samples from diseased plants collected in previous years. We observed that, among the different tissues evaluated, amplifications were consistent in samples from petioles, stems, and roots (Supplementary Table 3). While all primer sets amplified bands of the expected size, primer sets 1 and 3 also produced unspecific bands (*not shown*). Therefore, we proceeded with primer set 2 (CWBD-CIAT-F2/R2)*.* Next, we evaluated stem samples from 174 plants, with and without symptoms of CWBD, using primer set 2. We found a strong association between CWBD and the presence of *Ceratobasidium* sp. PCR bands of the expected size were obtained from all but one diseased plant, and in one asymptomatic plant, resulting in sensitivity and specificity values of 97.6% and 99.2%, respectively (Table 2, Supplementary Table 4). In all cases, the sequence of the PCR products corresponded to *Ceratobasidium* sp. isolates and showed nucleotide identities between 97.21 and 100% with the corresponding gene of *C. theobromae* (GenBank acc. no. KAB5596398) (Supplementary Table 3)*.* None of the samples yielded a PCR product when tested for phytoplasma infection using available nested PCR protocols.

**Table 2:** PCR analysis of field samples (stems from plants with and without disease symptoms) showed a strong association of *Ceratobasidium* sp. with CWBD.

Phenotype	Number of samples	PCR ( −)	PCR ( +)
Healthy	123	122	1
Diseased	41	1	40
Other symptoms*	3	0	0
Unclear symptoms**	4	0	0

Fields were surveyed following an X transect over a 1 ha field and samples were collected every 4th plant along the transects (see "Materials and Methods"). All PCR bands obtained were confirmed to correspond to *Ceratobasidium* sp. through sequencing. The sensitivity (97.6%) was calculated as TP/(TP + FN)*100, and specificity (99.2%) calculated as TN/(TN + FP)*100. TP, FN, TN, FP denotes True Negative, True Positive, False Negative, and False Positive, respectively. Additional data is available in Supplementary Table 4.

*Other symptoms included shoot tip dieback, small stems with lesions and yellowing.

**Unclear symptoms indicate plants with indistinguishable symptoms. Additional information in Supplementary Table 2.

### The causal pathogen of CWBD is transmissible by grafting

**Figure 5:**
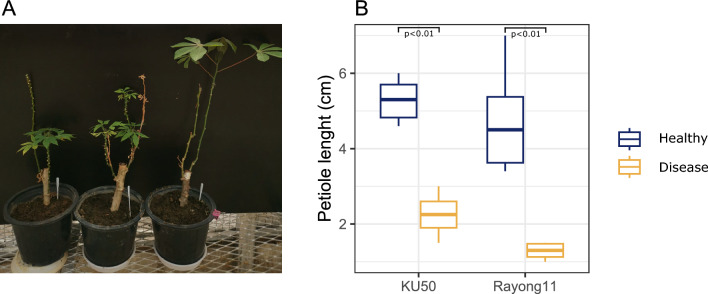
Induction of CWBD symptoms by grafting in cassava. (**A**) CWBD symptoms were induced after graft transmission of *Ceratobasidum* sp. to two cassava genotypes, KU50 and Rayong11. Left: an infected cassava plant; center: a healthy cassava plant grafted with an infected plant; right: a healthy cassava plant grafted with another healthy cassava plant. (**B**) Petiole length in healthy (blue) and diseased (yellow) plants during the experiment in both varieties. Box plots show significant differences (p < 0.05) according to the Wilcoxon non-parametric test.

The possibility of transmitting *Ceratobasidium* sp. was explored by grafting experiments. Several attempts at using chip bud grafting failed to transmit the pathogen over the course of the 6-months experiments. In our observations, this failure was attributed to the damage inflicted by CWBD on vascular tissue, impeding the establishment of young grafts. However, side grafting resulted in longer survival of the graft and was efficient in reproducing the symptoms, including leaf yellowing, shortened internodes, phyllody and reduced shoot height (Fig. 5A). We used the significant effect of CWBD on petiole length as measurable indicator of the disease severity (Fig. 5B). After 21 days, three out of three grafted Rayong 11 plants showed symptoms. In KU50 grafted plants, symptoms were apparent in two out of three plants after 50 days. All plants of genotype KU50 showed symptoms 5 months after grafting.

## Discussion

In this work, we used a metagenomic approach to analyze the microbial content of plants affected by CWBD. This was prompted by the unexpected proportion of false positives obtained when detecting phytoplasma in diseased plants^9^, an observation that is reflected in a lack of data on the efficiency of phytoplasma detection in CWBD-affected plants^4–8,19^. As described earlier, using total DNA sequencing, we readily identified a fungus of the *Ceratobasidium* sp. group, as the dominant microbe present in plants affected by CWBD, and no phytoplasma sequence was detected, (Table 1, Fig. 1). Results also show a dominance of *Methylobacteriaceae* in healthy plants and a significant decline in microbial diversity composition linked to CWBD (Fig. 1). Although similar cases have been reported for other diseases, whether these differences are a cause, or a consequence of disease remains unknown^20^; additional research is required before we can extrapolate such specific change in microbial diversity as a hallmark of CWBD.

Molecular analysis has been instrumental in fungi identification as it can resolve limitations associated with in vitro culture and the existence of morphological characteristics shared among different taxa^21–23^. In this case, although growing *Ceratobasidium* sp. in vitro could not be maintained over extended periods of time, its presence was confirmed by quick morphological and molecular analysis of hyphae samples obtained from diseased plants (Fig. 2)^24,25^. Genus-related features such as hyphal diameter, the presence of bi-nucleate vegetative cells, a right-angle branching pattern with a slight constriction at the branching point, and a dolipore septum near the branching point (Fig. 2)^24^, were observed. Phylogenetic analysis of the ITS region from isolates of Cambodia, Lao PDR, and Vietnam (Fig. 3) and PCR analysis from different tissues of CWBD-affected plants collected since 2012, further corroborated the presence and identity of the fungus in diseased plants and suggested petioles, stems or roots as target tissues for molecular diagnostics (Supplementary Table 3). Most interesting, these analyses identified the recently sequenced *C. theobromae*^16^*,* the causal agent of VSD in cacao^17^*,* as the closest relative of CWBD-associated *Ceratobasidium* sp. (Fig. 3)*.*

VSD is a disease that shares many characteristics in common with CWBD, including vascular necrosis and the formation of broom-like phenotypes with the characteristic accumulation of dry leaves^26^. *Ceratobasidium theobromae* is a near-obligate pathogen. It is a windborne, vascular pathogen, which establishes infection through germinating basidiocarps under wet and humid conditions. It has been shown that long-distance spread of VSD can be limited under low disease pressure^16^ and that symptoms of VSD appear late in the season^26^. CWBD shows a similar infection pattern in cassava^9^ and co-occur in SEA with VSD^9,18,26^. Further biological and genome analysis based on improved protocols for *Ceratobasidium* isolation^27^, should shed more light on the identity of both fungi. For example, it will be interesting to know whether *Ceratobasidium* sp. from cassava can infect cacao and produce related symptoms.

Another basidiomycetous fungus (*Moniliophthora perniciosa*) is known to cause witches’ broom and necrosis symptoms in cacao^28^. However, it appears geographically restricted to the Americas where the pathogen seems to have co-evolved with the host. *Ceratobasidium* sp. on the other hand, is likely to have jumped from a local host to the recently introduced cacao and cassava crops; and there is at least one report of the fungus infecting avocado^29^, another introduced crop, in Papua New Guinea. It is noteworthy that cacao and cassava can share additional pathogens, such as *Lasiodiplodia theobromae* and *Phytophthora palmivora,* causing root rot in both crops^30,31^. Given the economic and food security importance and ongoing commercialization of both crops in West Africa, SEA and the Americas, there is a considerable risk of movement of these understudied pathogens between regions and potentially other crops. Strict quarantine measures applied to the movement of planting material are crucial in reducing this risk.

Integrated management of CWBD, including the use of less susceptible genotypes, should contribute to disease control, but our results show that one of the most popular cassava genotypes grown in SEA (KU50), which is tolerant to CMD^15^, is susceptible to CWBD (Fig. 3). Tolerant varieties also carry the risk of inadvertently distributing the disease, moreover when effective and efficient diagnostic tools are lacking. In the case of CMD, alternative sources of resistance are under evaluation. However, resistance sources against CWBD are not yet found, and based on the increasing incidences of CWBD in the region (Fig. 4), they are urgently needed.

The PCR test described here shows robust results confirming the association of *Ceratobasidium* sp. with CWBD (Table 2; Fig. 5). Furthermore, it shows that the fungus has been present in the region since the first outbreaks of the disease (Supplementary Table 3). Its absence in material from the Americas (*not shown*), and its close relatedness to *C. theobromae* (Fig. 2) would further suggest a common geographical origin. Future research should map the diversity of *Ceratobasidium* isolates in the South Pacific, including the territories of Wallis and Futuna where the disease was reported for the first time^3^. Characterization of different strains of the fungus should include virulence tests and identification of genomic regions responsible for pathogenicity and virulence in different strains. This information should further improve diagnostics.

Interestingly, none of the samples analyzed by either DNA sequencing or by nested PCR, identified phytoplasma. It is unlikely that the CWBD outbreak observed during 2009–2012, and that currently observed are caused by different pathogens. Symptom records do not indicate any striking difference between both dates. Nevertheless, it is important to mention that we cannot discard the involvement of additional pathogens in the development or severity of CWBD symptoms; a co-infecting phytoplasma is a possibility and sequence data suggest the occurrence of up to 4 different ribosomal groups of phytoplasma occurring in SEA^8,9^. In any case, previous conclusions based on the role of phytoplasma infection in the diagnostics and biology of CWBD, without confirming the correlation between symptoms and identity of the pathogen^8,19^, should be taken with caution.

This is the first report of a *Ceratobasidium* sp. fungi associated with a cassava disease. Confirmation of *Ceratobasidium* sp. as the sufficient cause of CWBD remains to be proved. The fastidious nature of the fungus (i.e., requiring specific nutrients for in vitro culture) makes its maintenance in in vitro culture, and the production of infectious basidiospores a challenge that should be solved before Koch's postulates infections can be tested. In addition, we show that side grafting is a reproducible method to transmit the fungus and it could be implemented in screening for resistance assays as has been shown for other pathogens^32^. Together with an efficient low-cost PCR diagnostic now available, these are tools that will help accelerate rapid responses and facilitate basic research on this devastating disease of cassava.

## Materials and methods

### Plant material

All plant material collections and experiments were conducted in accordance to relevant institutional, national, and international permissions, guidelines and legislation, in coordination with our partner at the Plant Protection Center (PPC) of Lao PDR, the General Directorate of Agriculture (GDA) of Cambodia and the Plant Protection Research Institute (PPRI) of Vietnam. Plant material and photographs were collected during surveys in 2022 to determine the incidence of CWBD in cassava fields, 3–6 months after planting, in Lao PDR, Cambodia and Vietnam, as reported previously^33^. Plant samples were wrapped in paper towels and maintained dry in silica gel or were used fresh in the case of vascular stem tissue, before CTAB extraction^34^. Locations and photograph data were uploaded to our public repository PestDisPlace^35^. When testing the efficiency of PCR to detect the fungus, we evaluated samples from the top youngest leaves, root vascular tissue (RVT), stem vascular issue (SVT) and petioles from diseased leaves (Supplementary Table 3). For grafting, non–infected 9– to 10-month-old plants of KU50 and Rayong11 genotypes were collected in Naphok, Vientiane, Lao PDR from a field with no previous report of CWBD. For metagenomics analysis, DNA samples (264 ng on average) were extracted from fresh SVT of three diseased and three healthy plants, collected in the same field in Vientiane, Lao PDR.

### DNA extraction

DNA extraction from dry samples (20 mg of dry leaves or dry tissue, was carried out following the protocol described by Jimenez et al.^34^ while DNA extraction from fresh tissue (200 mg of stem or root vascular tissue) was carried out using a modified protocol. Briefly, collect 10 cm stem cuts from the central part (affected part) of a cassava stake of a 9– to 10-month-old plant and obtain the vascular tissue, which in CWBD-affected plants appears brown, by cutting the stake longitudinally, discarding the central part and scraping the vascular vessels next to the pith^36^. This vascular tissue was utilized for DNA extraction using CTAB^34^. The final nucleic acid pellets from all samples were air-dried for 15 min and eluted in 50 µl of nuclease-free water (Invitrogen). DNA was quantified using a Nanodrop 2000c spectrophotometer (ThermoFisher, USA) and visualized using 1% agarose gel electrophoresis. These samples were then stored at − 20 °C for future use. For PCR tests all nucleic acid extracts were adjusted to a concentration of 30 ng/µl, and 2 µl of the solution were used in a 25 µl reaction (see below). Total DNA extracted from different parts of the cassava plants collected in previous years and stored at − 20 °C was used to validate the occurrence of *Ceratobasidium* sp. in locations where the disease had been reported. For DNA isolation from hyphae, we used a modified miniprep protocol described by Raeder and Broda^25^. Briefly, two-week old fungal mycelium grown on PDA media was scraped from the petri dish and transferred to a 2 ml tube, the tubes were immersed in liquid nitrogen and the mycelium was then homogenized using plastic pestles. Subsequently, 200 µl of extraction buffer (15 mM NaCl, 50 mM Tris pH8.0, 10 Mm Na_2_EDTA, 1% [w/v] SDS) along with 1 µl of proteinase K (10 mg/mL) were added to the tube, and the mixture was vortexed until a homogeneous solution was obtained^37^. This solution was incubated at 65 °C for 1 h and the DNA purification process was continued as described^25^. The final DNA extract was diluted to a concentration of 30 ng/µl, and 2 µl of this solution was used for PCR amplification.

### Bioinformatics analyses

For metagenomics analysis, on average 250 ng of total DNA from three cassava plants with clear symptoms of CWBD and three healthy-looking plants was sequenced using the Illumina NovaSeq 6000 platform, provided by Fasteris LifeScience (Fasteris, Switzerland). The six datasets were obtained as fastq.gz files and deposited at the National Centre for Biotechnology Information. All sequence data underwent a rigorous analysis pipeline, including the following steps: Quality assessment and filtering were conducted using FastQC v0.11.9^38^ and Trimmomatic v0.39^39^ where reads with an average quality score < 30 were filtered out to ensure data quality. Reads that mapped to the cassava reference genome (GenBank acc no.GCA_001659605.2) were removed using BWA v0.7.17^40^. SAMtools v1.9^41^ was then used to extract the unmapped reads, which were subsequently assembled using MegaHIT^42^. The resulting contigs with a size ≥ 450 bp were taxonomically identified using DIAMOND^43^. To visualize DIAMOND results, the .daa files were uploaded to MEGAN6^44^ and then exported in BIOM table format to further data analysis in R v4.3.0. To further assess the microbial composition in both sets of samples (healthy and diseased), alpha diversity measures including the number of observed OTUs (richness), the Shannon index, and the Simpson index, were calculated. Subsequently, abundance was normalized by sequencing depth, and beta diversity was assessed with Bray–Curtis dissimilarity test, with principal coordinate analysis (PCoA) applied. Finally, the abundance was normalized with DESeq2^45^, and the Wald test was performed to determine significantly more represented genera in healthy and diseased plants.

### Primer design, PCR tests and phylogenetic analysis

A *Ceratobasidium* sp. contig with the highest coverage (> 20,000X) was used to design sets of PCR primers for specific detection of the fungus. This contig contained three Open Reading Frames (ORF) corresponding to a Hypothetical protein CTheo_14 (GenBank acc no. KAB5596377); a CAMK/CAMKL kinase (GenBank acc no. KAB5596398) and a hypothetical protein CTheo_35 (GenBank acc no. KAB5596385). One primer set per ORF was designed with Geneious® Prime software v2022.1.1 (Biomatters, New Zealand) using default parameters. The primer sets were named CIAT-CWBD-F1/R1 (736 bp), CIAT-CWBD-F2/R2 (1186 pb) and CIAT-CWBD-F3/R3 (686 bp). PCR tests were carried out in a Mastercycler® Gradient Thermal cycler (Eppendorf, USA), using 2X PCR GoTaq® Master Mix Green (Promega, USA). A total of 2 µl of plant total DNA (30 ng/µl) was used in a 25 µl volume reaction mixture that included 0.5 µl of each primer (10 µM) and MilliQ water. The PCR was conducted with the following thermal cycling conditions: an initial denaturation step at 95 °C for 5 min; followed by 35 cycles of denaturation at 95 °C for 40 s; annealing at 52 °C for 45 s, and extension at 72 °C for 1 min. All three primer sets yielded the expected size bands but sets 1 and 3 also produced unspecific bands of different sizes. Therefore, we continued the analysis with primer set 2: CWBD-CIAT-F2 5’-GGATGAGTTTAATCGCTCTAAC-3' and CWBD-CIAT-R2: 5’-GCGCTCTGGTGTTTCAAGTTTG-3'. This PCR primer set targets the coding region of a putative Ca^2+^/calmodulin-dependent protein kinase gene (CaMK) as identified in *C. theobromae* (GenBank acc no. KAB5596398). CaMK belongs to a class of Ser/Thr protein kinases that mediate Ca^2+^ signals to modulate diverse biological pathways^16^.

The sequence of all PCR products was validated through nanopore MinION sequencing (Oxford Nanopore Technologies, UK) following a standard protocol^46^. A subset of samples was also tested for phytoplasma, using a nested PCR protocol with primers P1A/P7A and R16F2N/R16R2^4,6,47^. In addition, *Ceratobasidium* sp. primers were subjected to testing against *Rhizoctonia solani* (isolated from *Brachiaria*)*, Fusarium oxysporum* (isolated from plantain) and *Colletotrichum* sp. (isolate from guava) to verify that there were no unspecific detections. For phylogenetic analysis at species level, we used the ITS ribosomal DNA (rDNA) primers ITS1/ITS4, ITS1 (5´-TCCGTAGGTGAACCTGCGG-3´) and ITS4 (5´-TCCTCCGCTTATTGATATGC-3´)^48^ to amplify an expected product of 580 bp sequence. The alignment and construction of the phylogenetic tree were performed using Maximum Likelihood based on GTR + G model (1000 replicates) with related sequences available in GenBank. The evolutionary distances were computed using the Maximum Likelihood method and Kimura 2-parameter model. The heuristic search was obtained by applying Neighbor-Joining and BioNJ algorithms to a matrix of pairwise distances estimated using the Maximum Composite Likelihood (MCL) approach. There were 646 positions in the final dataset. All positions containing gaps and missing data were eliminated. These evolutionary analyses were conducted using MEGA11 v.11.0.13 and FigTree v1.4.4.

### Microscopical observations

Fungal specimens were isolated from petioles measuring 3–4 cm in length, which were previously disinfected by immersing them in a 1% hypochlorite solution for 1.5 min and in 75% ethanol for 2 min. The ethanol was removed by rinsing using sterile distillate water for 3 min and the petioles were dried in sterile tissue paper. These disinfected petioles were then transferred into potato dextrose agar (PDA) and incubated at 25 °C for 5 days, or they were promptly transferred to fresh PDA media if signs of contamination appeared^27^. All steps were performed in a laminar flow hood. For microscopical observation, the fungal hyphae were treated with 1% Methylene Blue solution (Merk, Germany) to facilitate the examination of morphological characteristics of the group. To assess the nuclear condition, the hyphae were stained using a Safranin O (Merk, Germany) standard solution (79 ml water, 6 mL 0.5% Safranin, 10 mL 3% KOH, 5 mL glycerin). Small white sclerotia (mycelia) samples were placed on a microscope glass slide and treated with 3% KOH for 40 min, then Safranin O solution was added, and the slides were incubated at room temperature for 20 min before placing the coverslip^49^. Samples were observed with an optical light microscope (Leica ICC50 HD, Germany) at 400 × magnification. The identity of the sample was confirmed by nanopore sequencing of PCR products^46^, using the *Ceratobasidium* sp. specific primers described above.

### Graft transmission

Grafting using axillary buds (chip bud grafting) and mature stakes (side grafting) was conducted to assess the transmission of CWBD with cassava genotypes KU50 and Rayong11 as rootstocks. These experiments were carried out at the Plant Protection Center in Vientiane and in screenhouse facilities of CIAT's Cassava Program at the Rice and Cash Crops Research Centre, in Naphok, Vientiane. For side grafting a shallow slit was made 1.5 cm above the 3rd or 4th internode of rootstocks using a sterile scalpel blade (Merck, Germany). A matching slice was made on the 2-node scion without leaves. The scion was then inserted in the incision on the rootstock and the combination was tightly wrapped with parafilm. The parafilm was removed 10 days post-grafting without damaging the graft union. Chip-bud grafting was carried out as previously described^50^. Healthy stakes were collected from 9– to 10-month-old plants grown in a research field where no CWBD had been observed. Three stakes from each genotype were used as rootstocks when they were 1-month after being transferred to a screenhouse. Rootstocks were pruned right before grafting and the grafted regions were wrapped with parafilm. Scions were collected from 9–10-month-old plants showing clear symptoms of CWBD. The plants were maintained under high humidity conditions (covered with plastic bags) for 6 days after grafting. Grafted plants were watered once a week and ten days post-grafting the parafilm was removed. Rootstocks were pruned again 1.5 months after grafting to stimulate new growth. Data was collected every week. The experiment was conducted twice, first from September 2022 to January 2023 and a second trial during October 2022 to February 2023.

### Field monitoring and association studies

To measure the incidence of CWBD in the region, field surveys were co-organized with partners from National Plant Protection Offices in Lao PDR (Plant Protection Center), Cambodia (General Directorate of Agriculture) and Vietnam (Plant Protection Research Institute). Sixty plants per hectare were inspected with each plant undergoing the collection of a biological sample along with its corresponding photographs^9,33^. The survey incidence data showed a non-parametric distribution, as confirmed by the Kolmogorov–Smirnov test. Consequently, we used the Wilcoxon non-parametric test to assess significant differences between the results obtained in 2020 and 2022. A subset of samples from the surveyed fields in Lao PDR (174 plants) was selected to examine the association between CWBD symptoms and the presence of *Ceratobasidium* sp. (Table 2) using the specifically design *Ceratobasidium* sp. PCR primers described above (CIAT-CWBD-F2/R2) using SVT samples from fields where cassava was 6 months old (> 6 months after planting). We also re-analyzed DNA samples extracted from plants collected in previous years from locations with high incidences of CWBD (Supplementary Table 3). In parallel, these samples were also evaluated for phytoplasma using published nested PCR protocols^4,6,47^. All amplified PCR bands of the expected size were purified and sequenced, to confirm their identity.

### Supplementary Information

Supplementary Table 1.

Supplementary Table 2.

Supplementary Table 3.

Supplementary Table 4.

## Data Availability

The sequences reported in this study have been deposited in the NCBI database with BioProject PRJNA973101 and BioSample SAMN35676008 to SAMN35676013. The data presented in this study are available in the following Supplementary Materials section.
